# Supplementary material for: Layered double hydroxide membrane with high hydroxide conductivity and ion selectivity for energy storage device
Source: Nat Commun. 2021 Jun 7;12:3409. doi: 10.1038/s41467-021-23721-9 (PMC8184958; doi:10.1038/s41467-021-23721-9)
Supplement: Supplementary file 1 — Supplementary Information [file 41467_2021_23721_MOESM1_ESM.pdf]

Supplementary Information For

Layered double hydroxide membrane with high hydroxide conductivity and ion selectivity for energy storage device

by Hu et al.

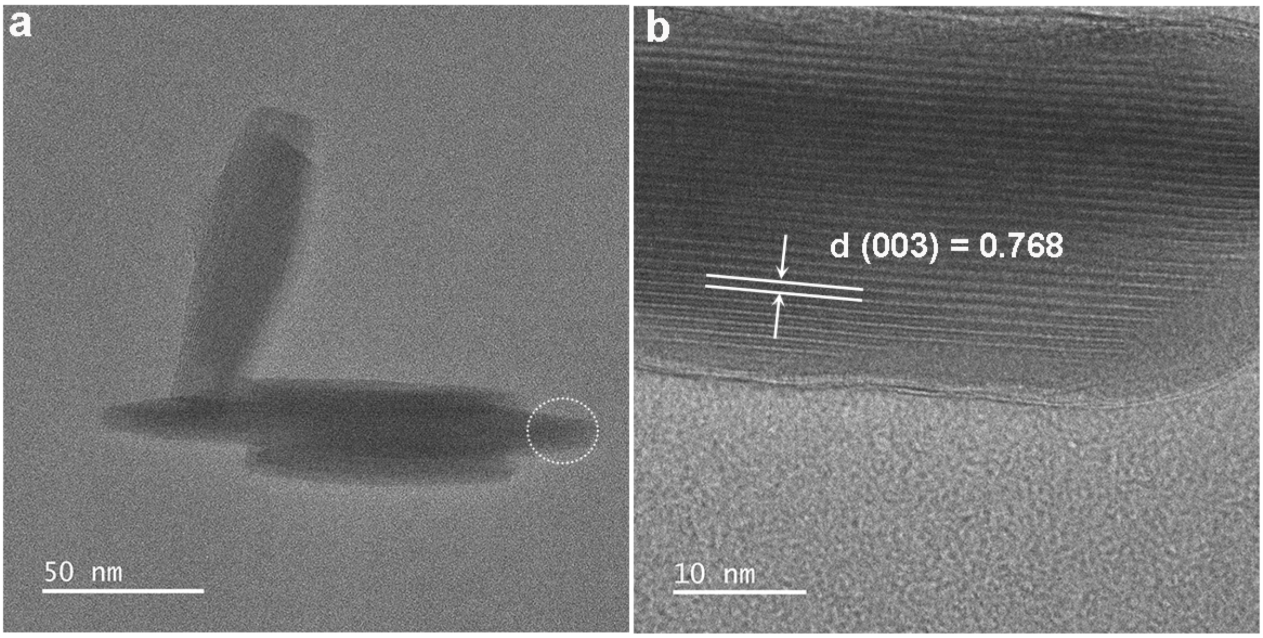

Supplementary Figure 1. The environmental transmission electron microscopy (ETEM) images of LDHs. a, The ETEM image of LDHs. **b,** The high resolution ETEM image of the area circled by the white frame in **a**.

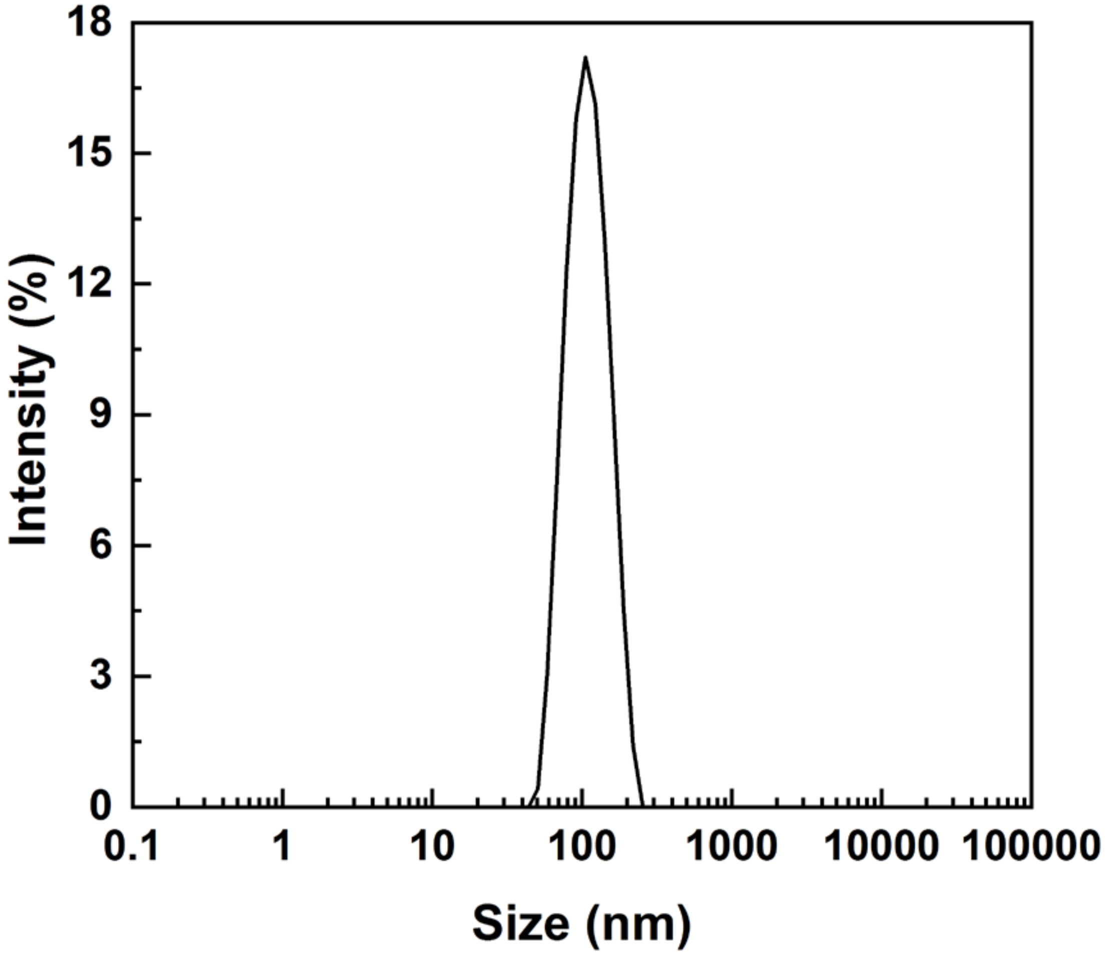

Supplementary Figure 2. Particle size distribution of LDHs.

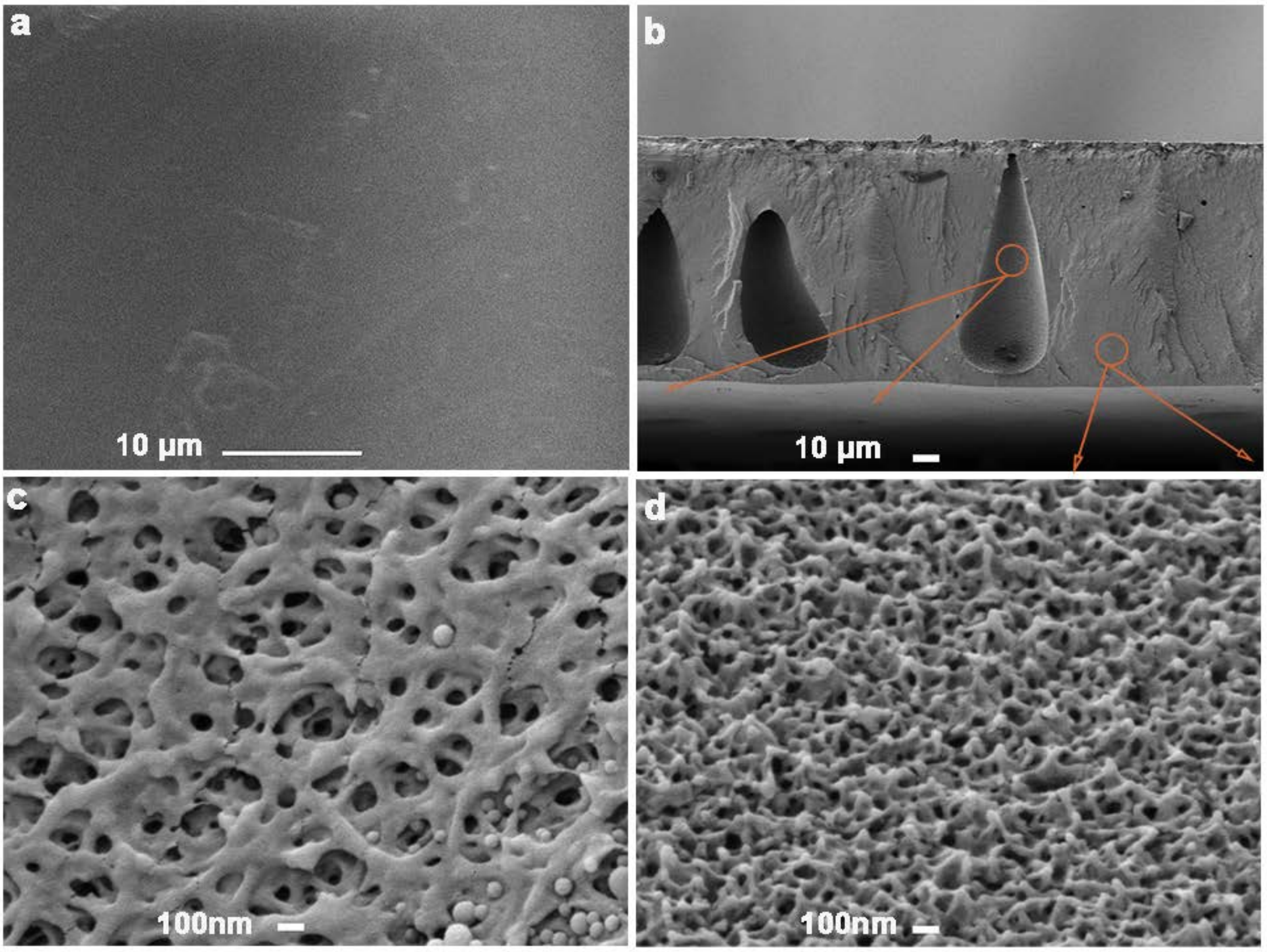

Supplementary Figure 3. SEM images of substrate membrane. a, Surface morphology of substrate. **b,** Cross-section morphology of substrate. **c, d** The magnified morphologies of the circled area in **b**.

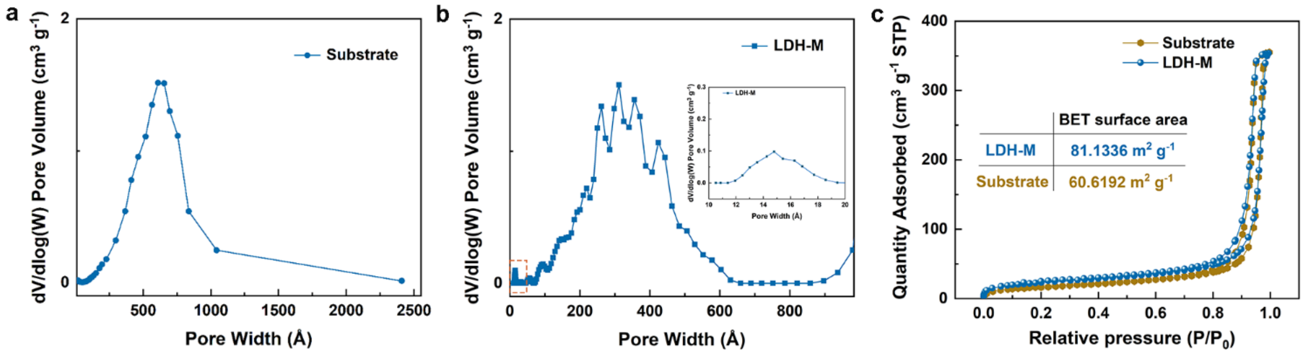

Supplementary Figure 4. The BET results of LDH-M and substrate. a, The pore size distribution curve of the substrate**. b,** Pore size distribution curve of LDH-M**. c,** N_2 isothermal absorption/desorption curves of LDH-M and substrate**.**

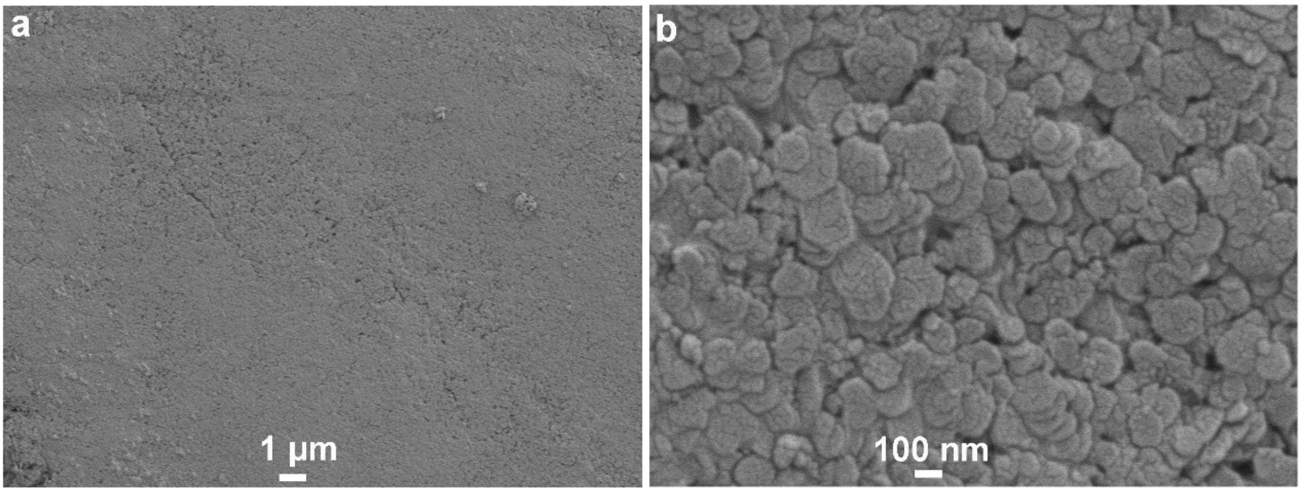

Supplementary Figure 5. SEM images of LDH-M membrane. a, Surface morphology of LDH-M membrane. **b,** magnified surface morphology of LDH-M membrane.

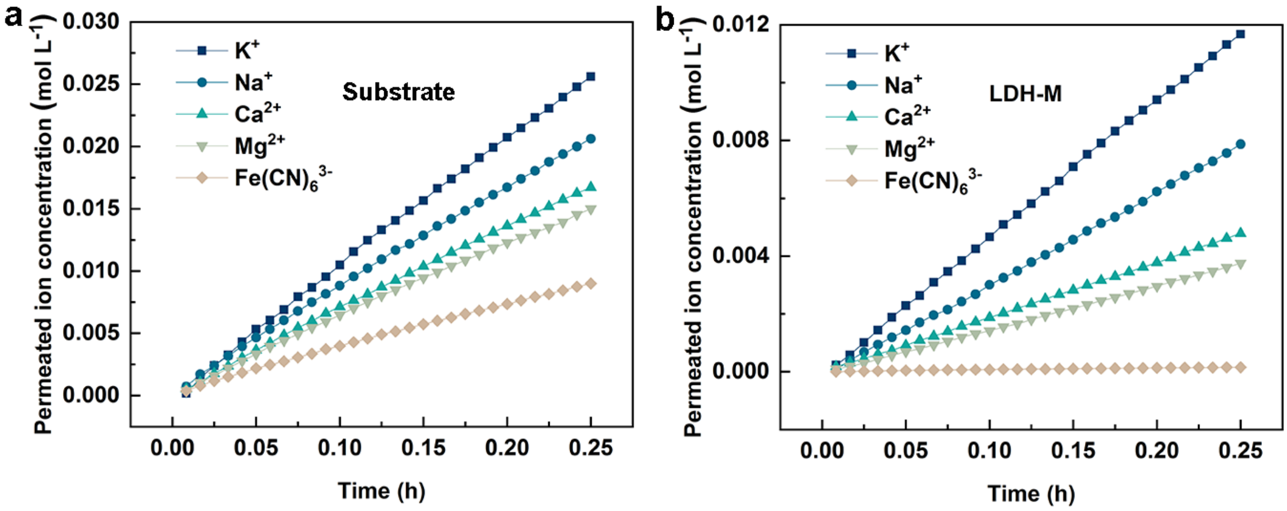

Supplementary Figure 6. The permeability of cationic ions and $Fe(CN)_6^{3-}$. Comparison of cationic ions and $Fe(CN)_6^{3-}$ for **a,** substrate membrane and **b**, LDH-M membrane.

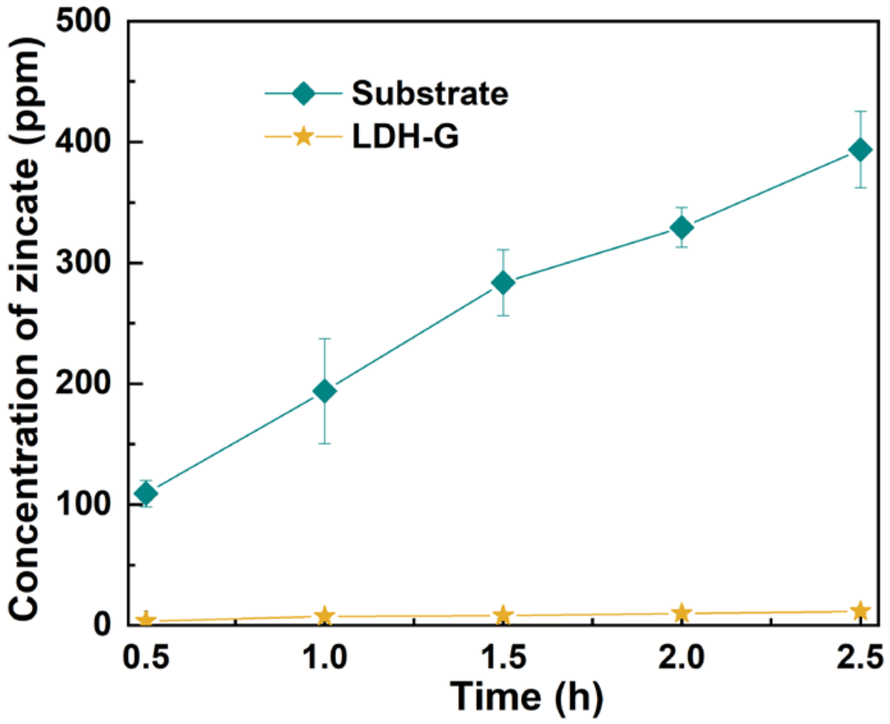

Supplementary Figure 7. The permeability of zincate ion for LDH-M and substrate. Error bars are standard deviations using at least three measurements from different samples.

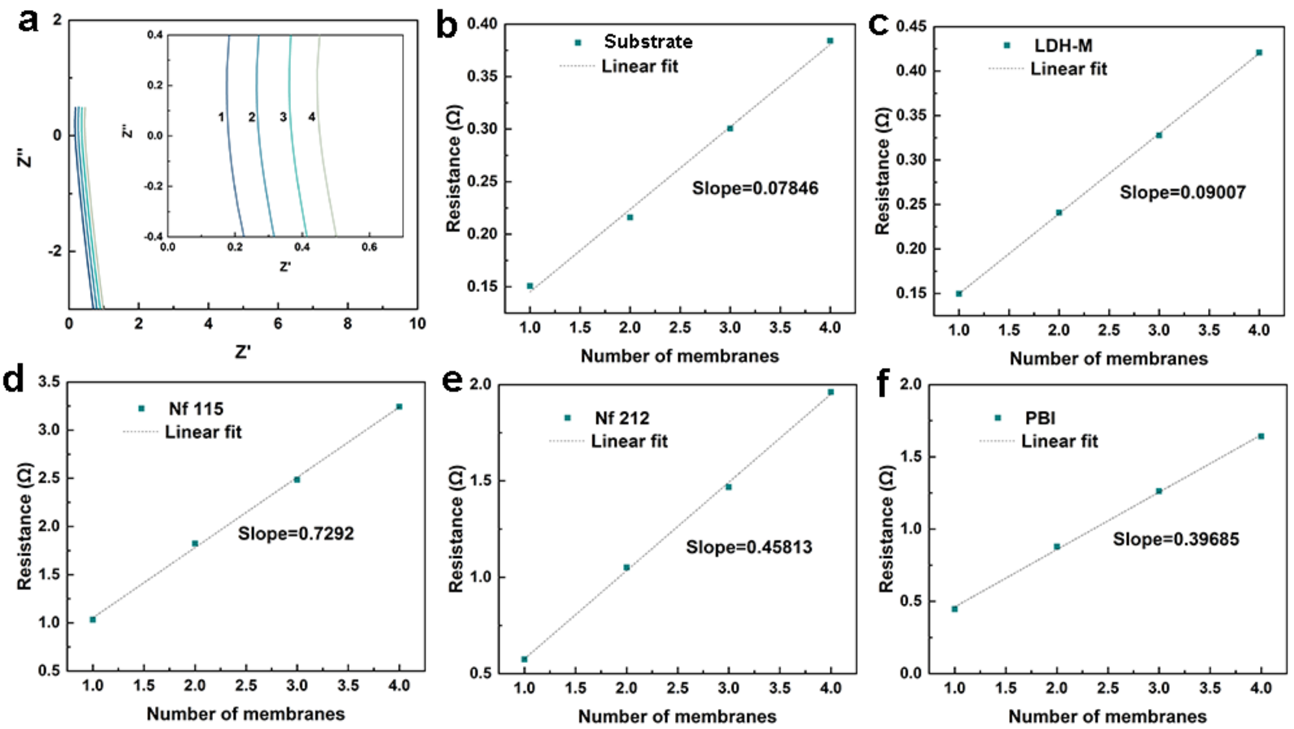

Supplementary Figure 8. The membrane resistance measured by EIS in 3 mol L^{-1} sodium hydroxide solution. **a,** The EIS plot of different numbers of LDH-M stacked layer-by-layer. Inset: The magnified EIS plot, The EIS plot shift to right with the number of membrane increases. **b,** The change of the resistance with the numbers of substrate. The resistance of one membrane was calculated from the slope of the linear fit of the resistance vs. the numbers of membranes stacked layer-by-layer. As a result, the membrane resistance is 0.07846 Ω for this sample. The conductivity and area resistance of a membrane is calculated by equation (1). The effective area is 1.766 cm^2 and the thickness of substrate is 105 μm measured with a micrometer caliper. As a result, the conductivity of substrate is 0.076 S cm^{-1} and the area resistance is 0.138 Ω cm^2. c,d,e,f, The change of the resistance with the numbers of LDH-M (c) Nf115 (d) Nf212 (e) and PBI (f) . The membrane conductivity of the membranes were measured and calculated with the same procedure as substrate.

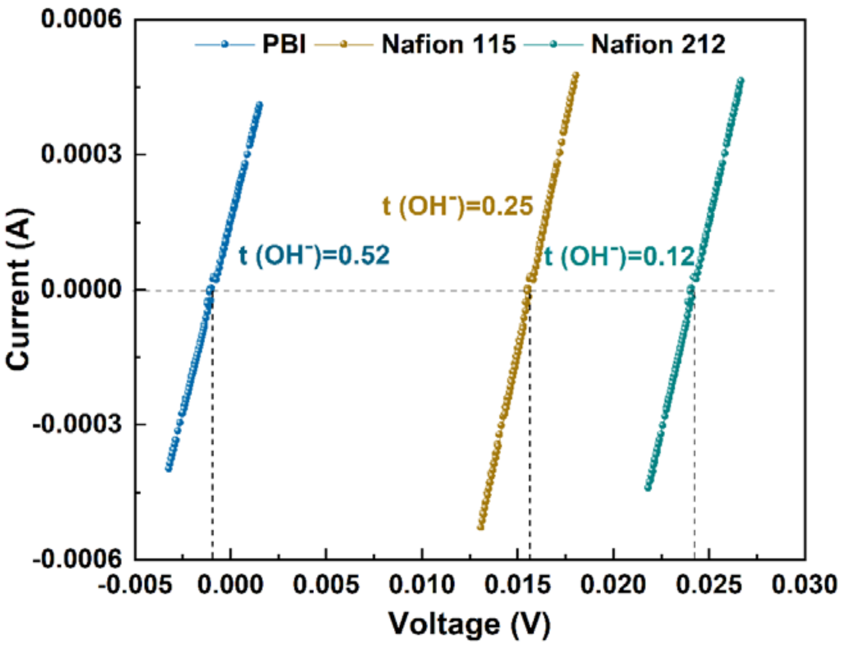

Supplementary Figure 9. The hydroxide ion transference numbers through different membranes calculated from the current-voltage (I-V) profiles.

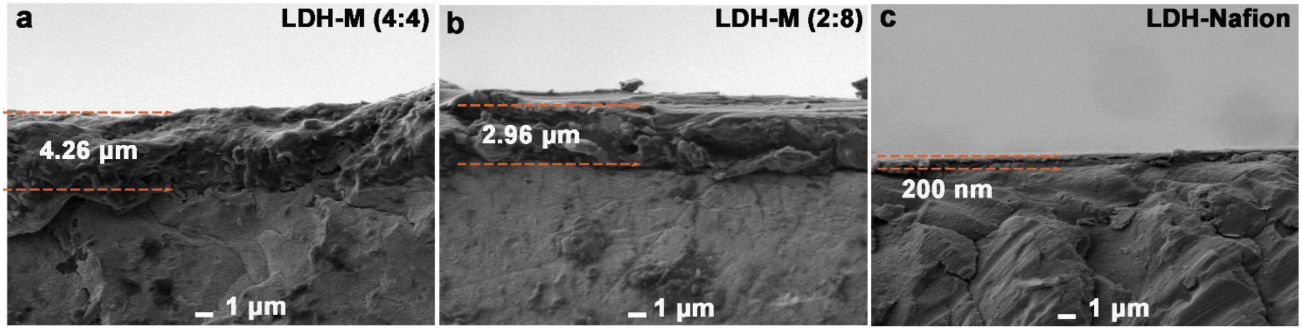

Supplementary Figure 10. The cross-section morphology of prepared membranes with different ratio of LDHs/Nafion. **a**, LDH-M (4:4). **b**, LDH-M (2:8). **c**, LDH-Nafion (0:1).

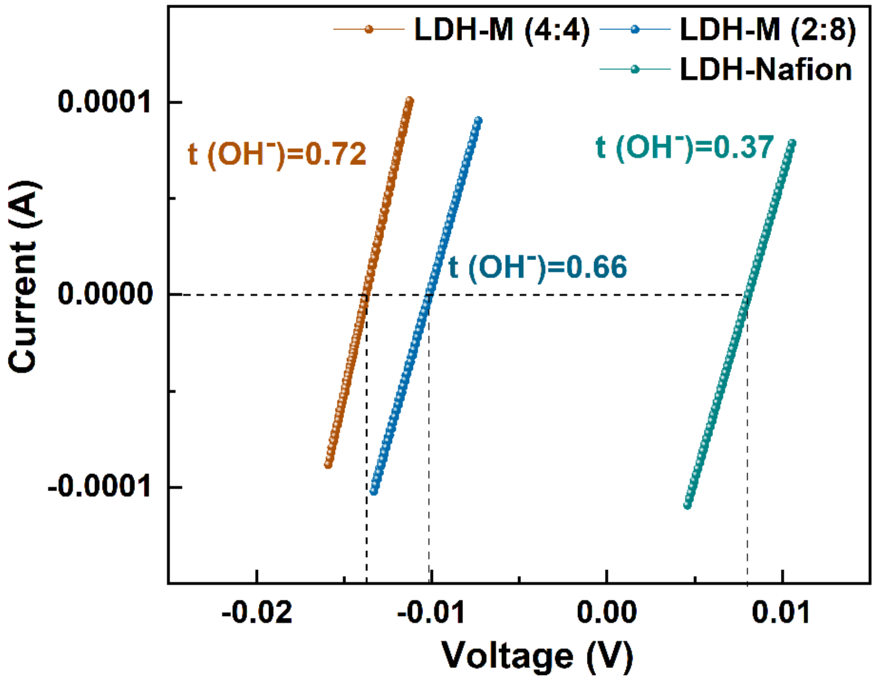

Supplementary Figure 11. The hydroxide ion transference numbers through different membranes (prepared from different ratio of LDHs/Nafion) calculated from the current-voltage (I-V) profiles.

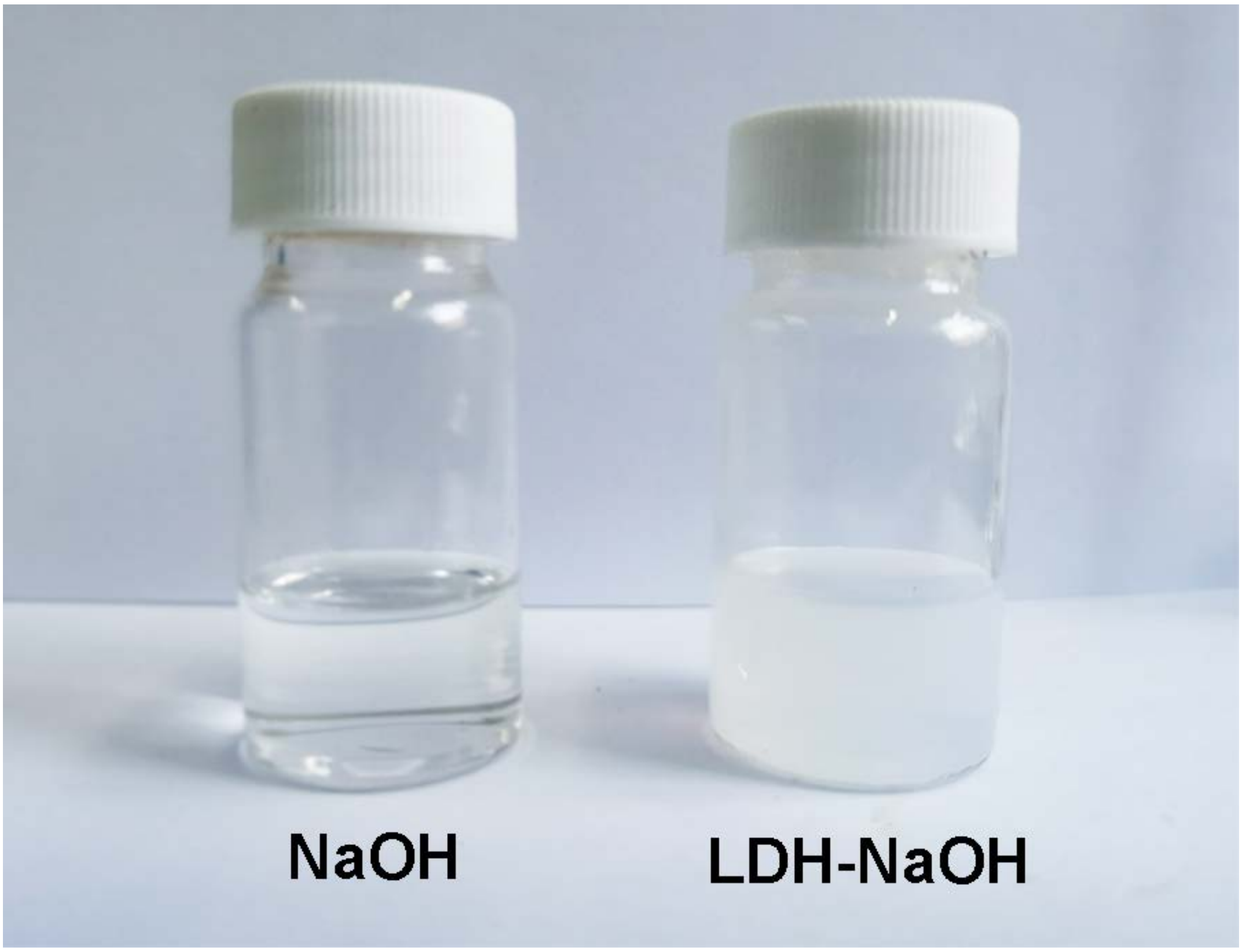

Supplementary Figure 12. The visual experiment of the white precipitate of silver chloride verifies that the chloride ion is exchanged after soaking MgAl-Cl-LDH in 3 mol L^{-1} NaOH solution. The pure 3 mol L^{-1} NaOH solution supernatant, by adjusting the pH to acidity with 3 mol L^{-1} nitric acid, then adding 1 mol L^{-1} $AgNO_3$ into above solution, finally no white precipitation of AgCl was observed. In contrast, taking the NaOH supernatant which soaked with MgAl-Cl-LDH, by adjusting the pH to acidity with 3 mol L^{-1} nitric acid, then adding 1 mol L^{-1} $AgNO_3$ into above solution, finally a white precipitate appears, which proves that there exists exchanged Cl^{-} in the supernatant.

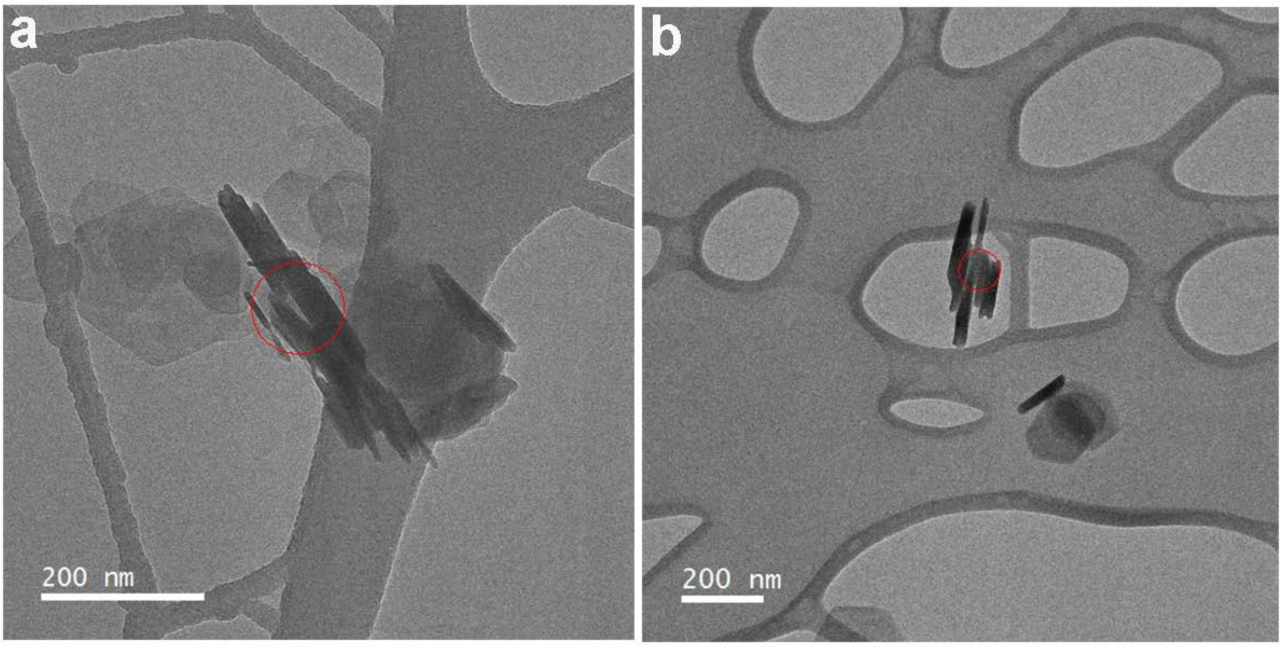

Supplementary Figure 13. The ETEM images of prepared samples. The red cycle represent the selected area for (003) plane diffraction of **a**, MgAl-Cl-LDH and **b**, MgAl-OH-LDH.

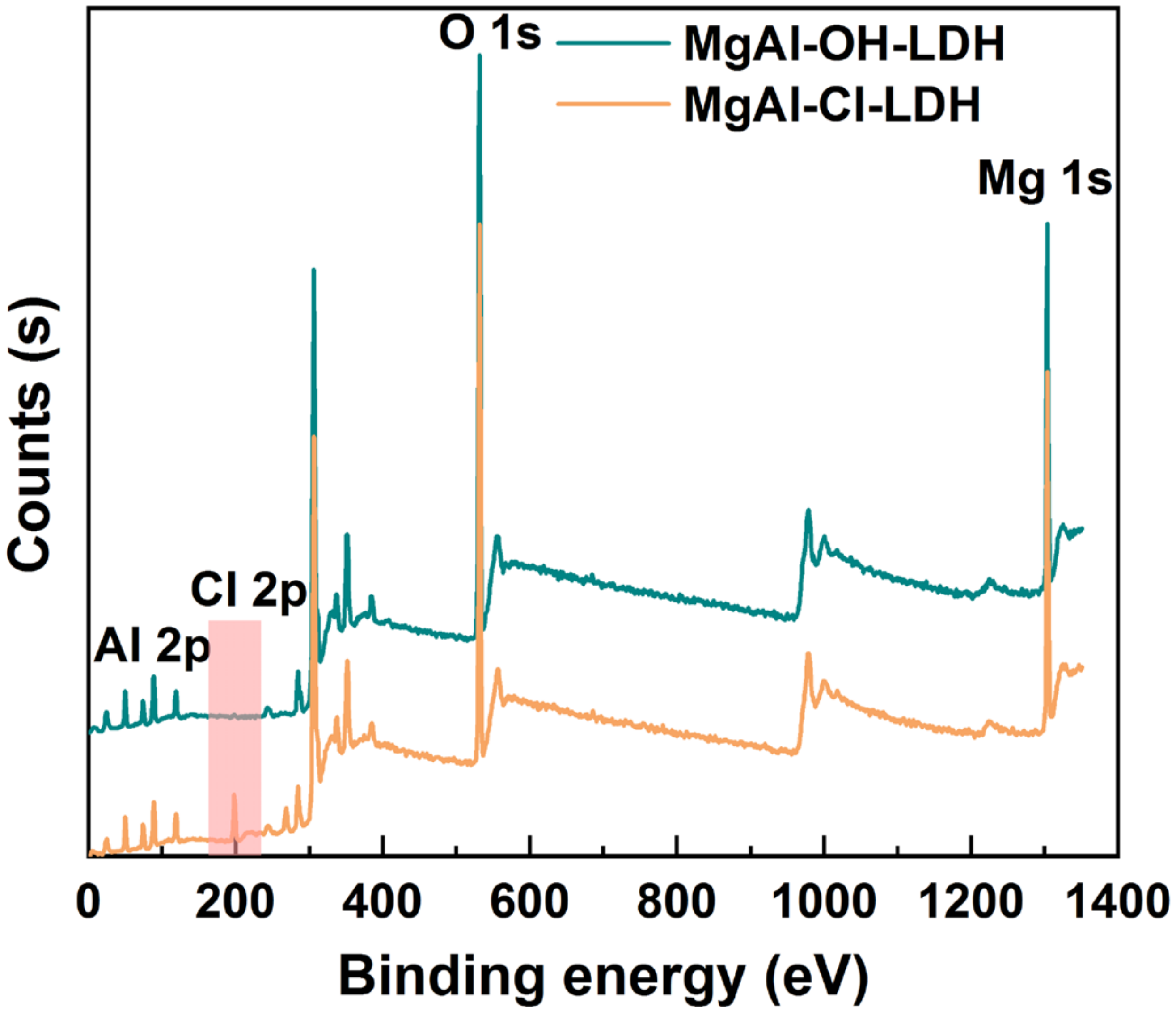

Supplementary Figure 14. The XPS survey spectra of MgAl-Cl-LDH and MgAl-OH-LDH samples. Note that the disappearance of Cl element could be observed for MgAl-OH-LDH sample.

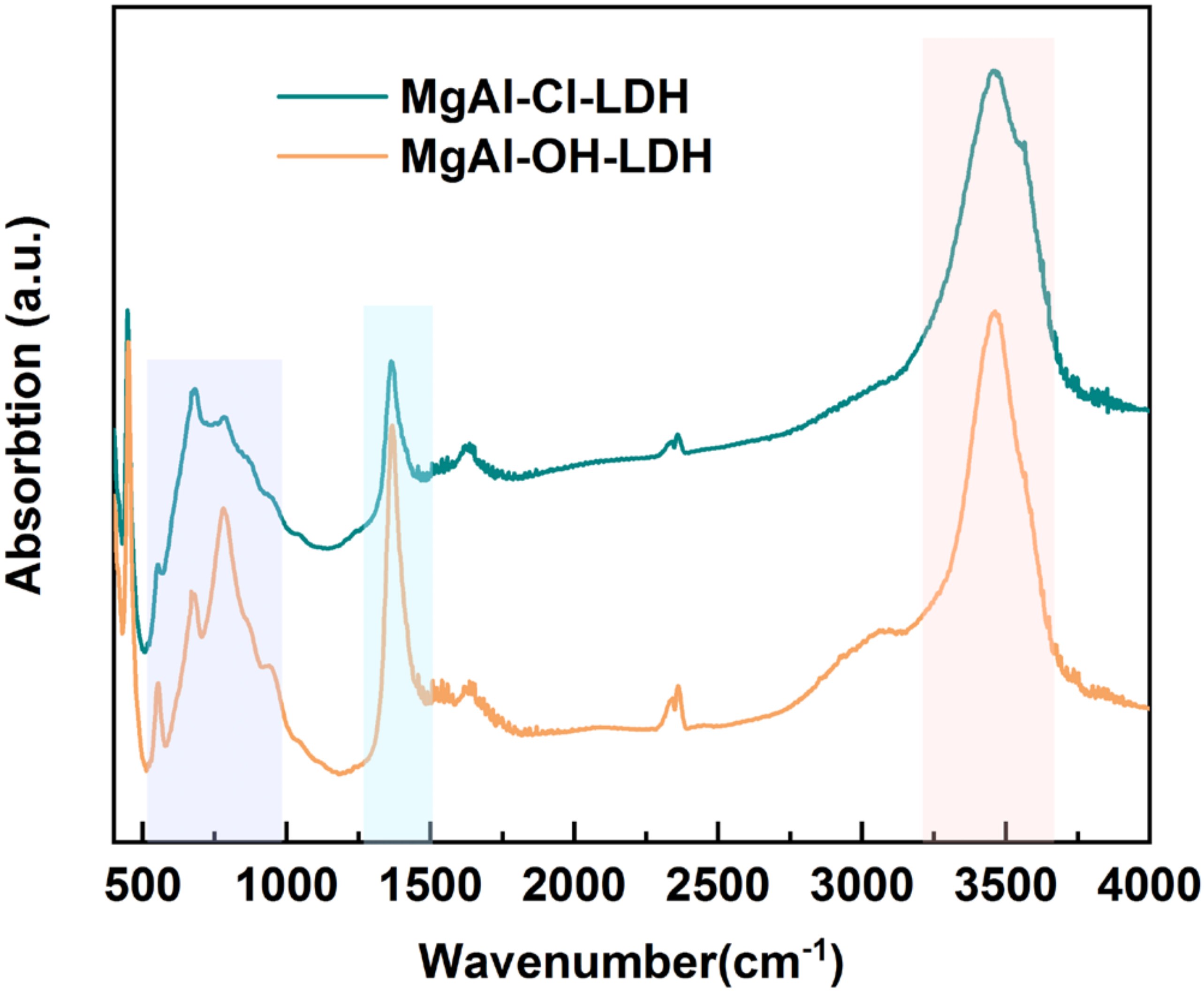

Supplementary Figure 15. The FT-IR spectra of prepared MgAl-Cl-LDH and MgAl-OH-LDH samples.

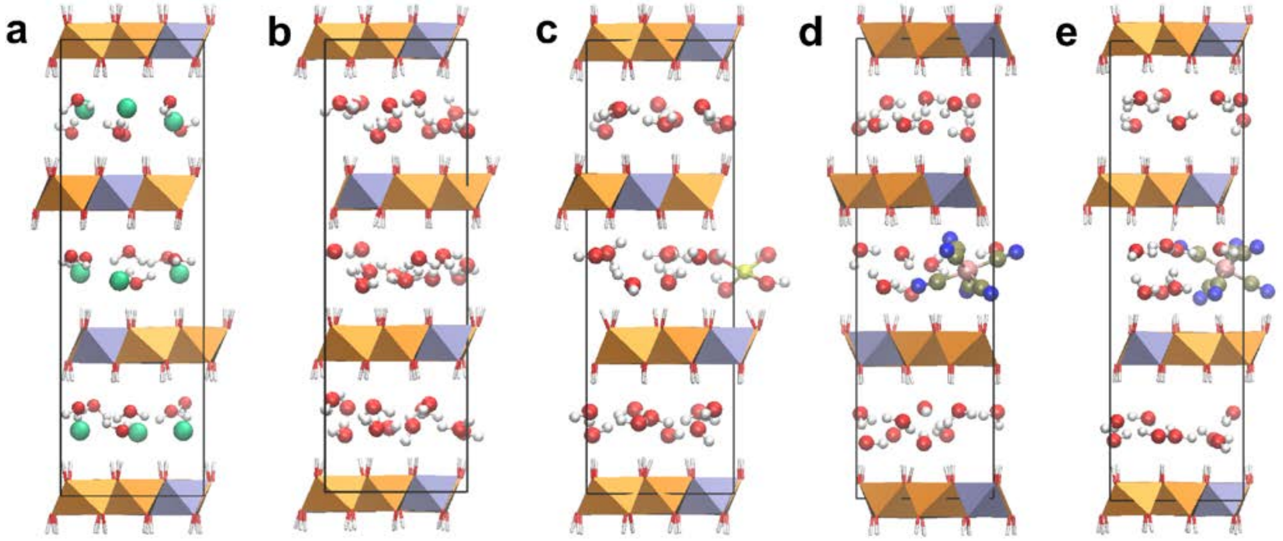

Supplementary Figure 16. Optimized structures for layered double hydroxide. a, model-0 (3 × 3 × 1 supercells of $Mg_2Al(OH)_6Cl{\cdot}2H_2O$); **b,** model-1 (model-0 structure with Cl^- substituted by OH^- with the addition of two water molecules per layer), **c,** model-2 (model-0 structure with two Cl^- substituted by one $Zn(OH)_4^{2-}$ and other Cl^- substituted by OH^-), **d** model-3 (model-0 structure with three Cl^- substituted by one $Fe(CN)_6^{4-}$ and other Cl^- substituted by OH^-) and **e** model-4 (model-0 structure with four Cl^- substituted by one $Fe(CN)_6^{4-}$ and other Cl^- substituted by OH^-**).** Mg atoms of hydrotalcite layer were shown in orange octahedra, Al atoms of hydrotalcite layer were shown in ice blue octahedra, Cl anions were shown in green balls, Zn atom was shown in yellow ball, Fe atom was shown in pink ball, N atoms were shown in blue balls, C atoms were shown in tan balls, O atoms in hydroxide ions and water molecules were shown in red balls, O atoms of LDHs were shown in red stick, H atoms in hydroxide ions and water molecules were shown in white balls, and H atoms of LDHs were shown in white sticks, respectively.

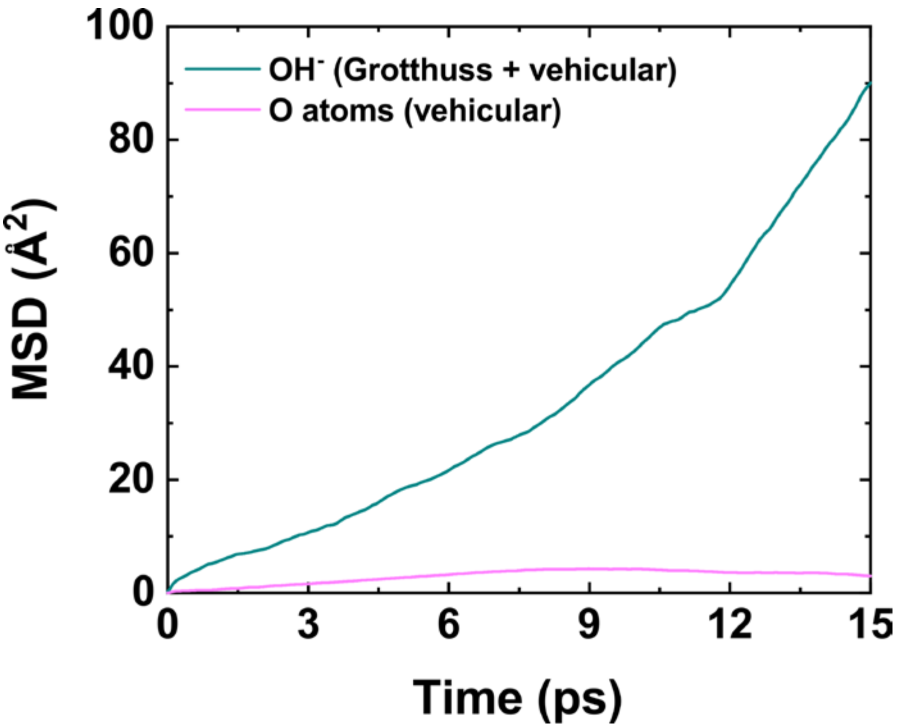

Supplementary Figure 17. Mean square displacement (MSD) of hydroxide ions which accounts for the movement induced by both Grotthuss and vehicular mechanisms, as well as MSD of all O atoms in water and hydroxide ions which only represents contribution of vehicular mechanism in LDHs at 298 K.

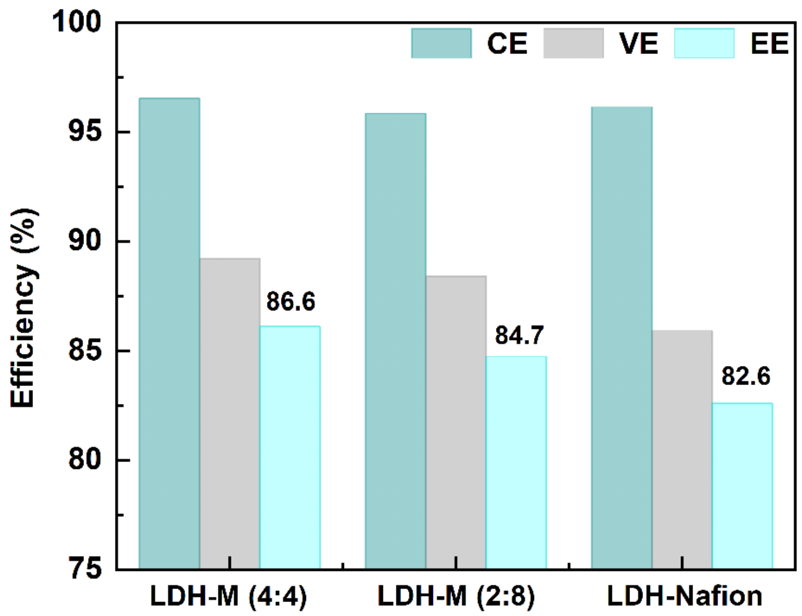

Supplementary Figure 18. The AZIFB performances assembled with different membranes at the current density of 80 mA cm^{-2}.

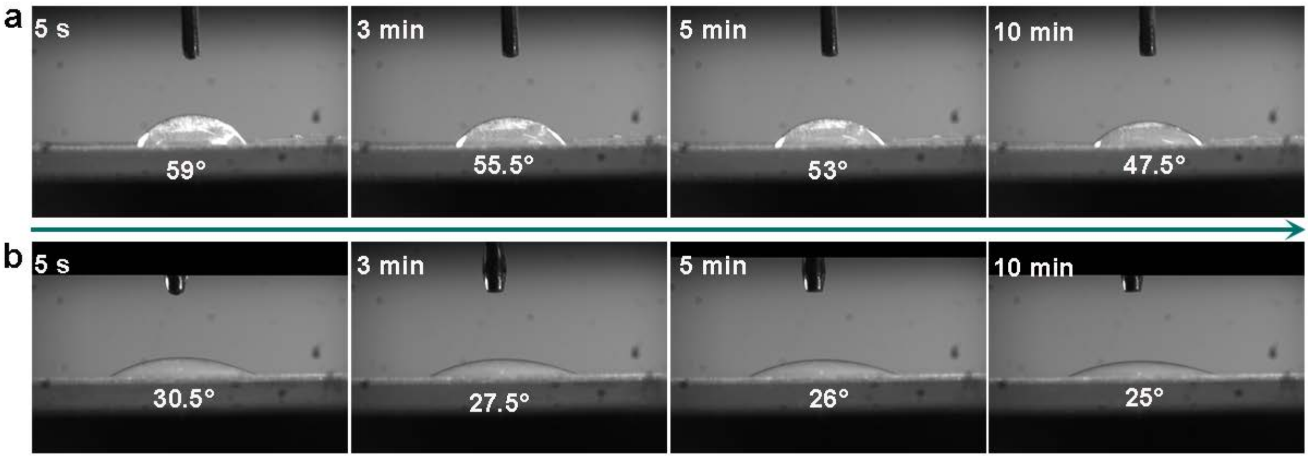

Supplementary Figure 19. Contact angle of prepared membranes using 3M NaOH as feeding solution. Contact angle of **a,** substrate and **b,** LDH-M membrane. As time processes, the contact angle values decrease, while LDH-M shows better wettability with NaOH solution than substrate.

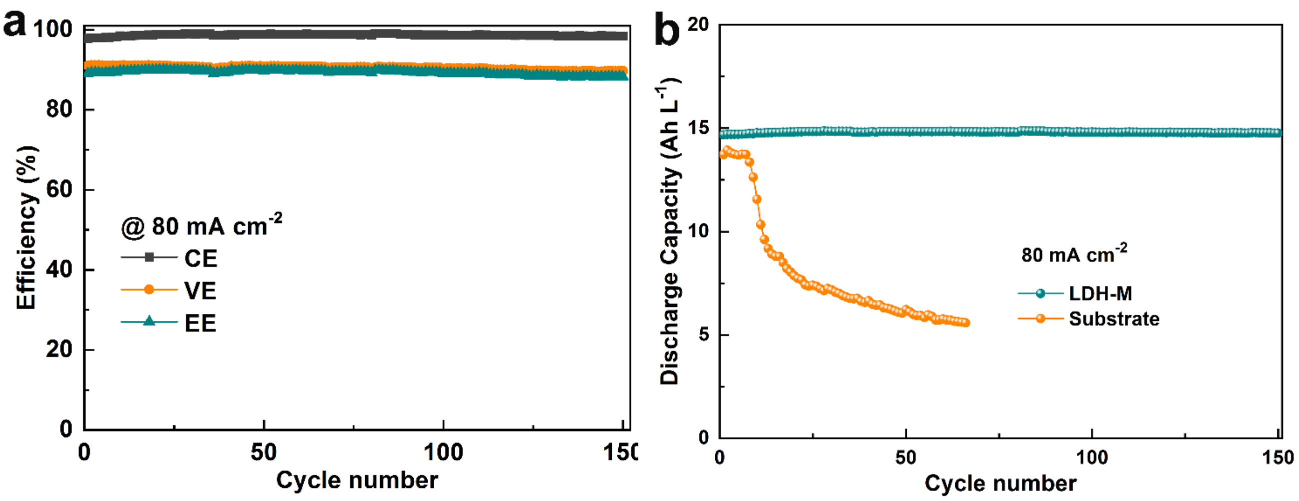

Supplementary Figure 20. Electrochemical performance of the alkaline zinc-iron flow battery employing LDH-M membrane and substrate at the current density of 80 mA cm^{-2}. a, Cycling performance of AZIFB with LDH-M. **b,** The comparison of discharge capacity for AZIFB with LDH-M and substrate.

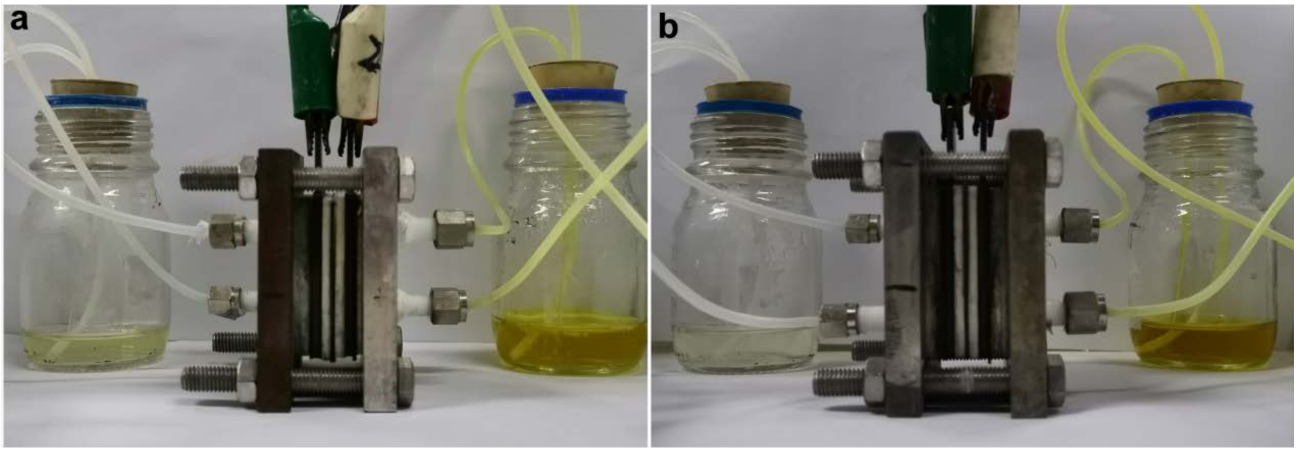

Supplementary Figure 21. The electrolytes cross over images of alkaline zinc iron flow battery with substrate and LDH-M at the current density of 80 mA cm^{-2}. a, The negative and positive electrolytes image at the end of 5th discharge for AZIFB with substrate. The obvious color change of negative electrolyte can be found, indicating the cross-over of the electrolyte. **b,** The negative and positive electrolytes image at the end of 5th discharge for AZIFB with LDH-M.

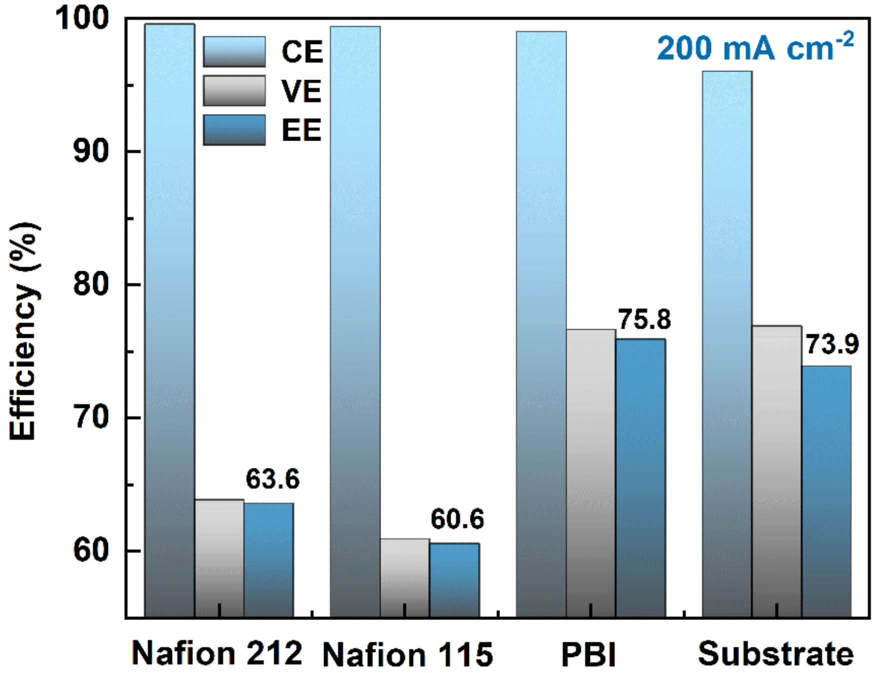

Supplementary Figure 22. Efficiencies of AZIFBs equipped different membranes at the current density of 200 mA cm^{-2}.

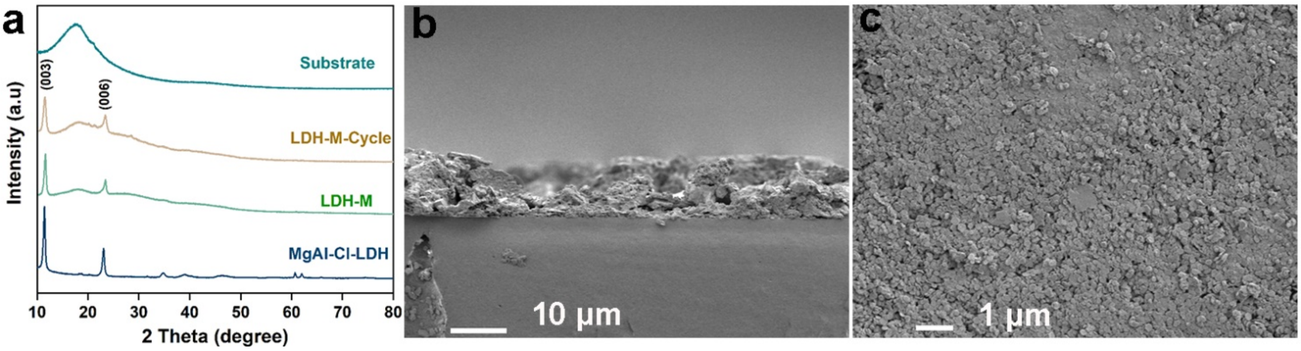

Supplementary Figure 23. The structures of the LDH-M membrane after nearly 400 cycles charging-discharging test at the current density of 200 mA cm^{-2} (LDH-M-Cycle). a, The XRD patterns of MgAl-Cl-LDH, Substrate, LDH-M and LDH-M-Cycle. **b,** The cross-section and **c**, surface morphology of LDH-M-Cycle**.**

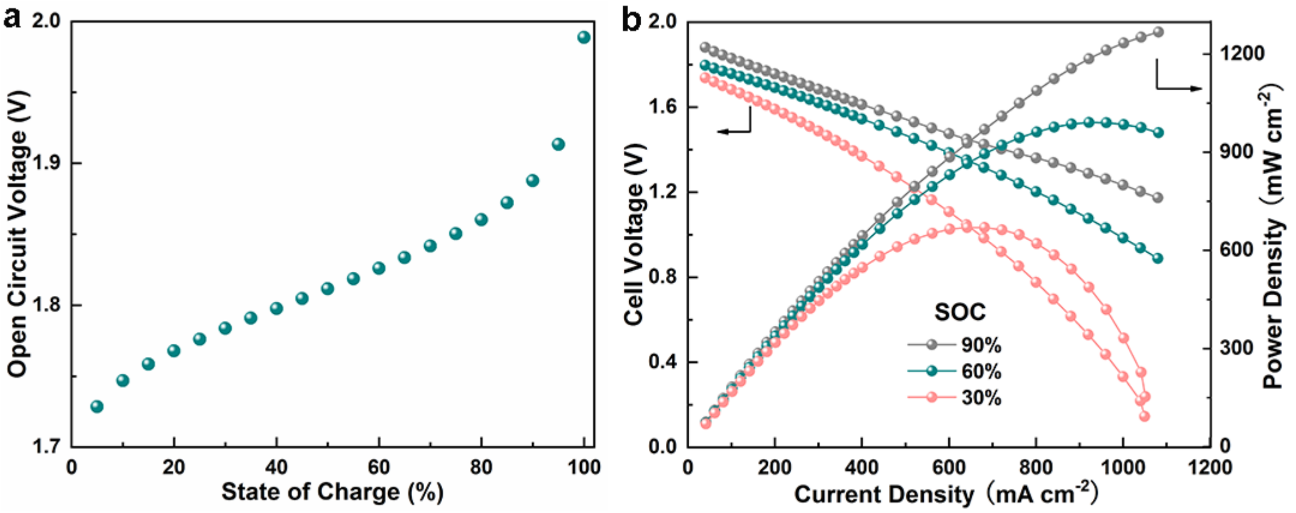

Supplementary Figure 24. Electrochemical performance of the alkaline zinc-iron flow battery employing LDH-M membrane. a, The open-cell voltage (OCV) versus state-of-charge (SOC) of AZIFB. **b,** The polarization curves of AZIFB at 90% SOC, 60% SOC, and 30% SOC.

Supplementary Table 1. Battery performance of recently reported zinc-based flow battery systems.

Aqueous flow battery	Membrane	Current density (mA cm^{-2})	Performance EE(%)	Cycles
Zn/Br_2[1]	polyolefin porous (PP)	160	80	100
Zn/I_2[2]	PP/Nafion	80	72.8	500
Alkaline Zn/I_2[3]	Nafion117	20	70	10
Zn/TEMPO[4]	fumasep F-930-RFD	80	50	—
Zn/Fe[5]	Nafion	80	61.5	100
Neutral Zn/Fe[6]	Microporous	25	68	120
Neutral Zn/Fe[7]	Porous PBI	80	78	100
Alkaline Zn/Fe[8]	Nafion212	80	76	20
Alkaline Zn/Fe[9] (our previous work)	Nafion 212	80	80-85	100
Alkaline Zn/Fe[10] (our previous work)	PES/SPEEK	160	80	100
Alkaline Zn/Fe[11] (our previous work)	PBI	160	82.68	150
**This work**	**LDH-G**	**200**	**82.36**	**400**

Supplementary Table 2. Mechanical performance of different membranes.

Membrane	Elongation at break (%)	Breaking stress (MPa)	Tensile strength (MPa)
Nafion 212	83.11	24.58	24.58
Nafion 115	119.28	20.36	20.36
Substrate	37.56	12.98	13.13
LDH-M	37.94	12.19	12.36
LDH-M-Cycle	33.72	12.12	12.16

Reference

1 Wang, C. et al. A TiN Nanorod Array 3D Hierarchical Composite Electrode for Ultrahigh-Power-Density Bromine-Based Flow Batteries. *Adv. Mater.*, e1904690, doi:10.1002/adma.201904690 (2019).

2 Xie, C., Liu, Y., Lu, W., Zhang, H. & Li, X. Highly stable zinc–iodine single flow batteries with super high energy density for stationary energy storage. *Energy Environ. Sci.* **12**, 1834-1839 (2019).

3 Zhang, J. et al. An all-aqueous redox flow battery with unprecedented energy density. *Energy Environ. Sci.* **11**, 2010-2015 (2018).

4 Winsberg, J. et al. Aqueous 2,2,6,6-Tetramethylpiperidine-N-oxyl Catholytes for a High-Capacity and High Current Density Oxygen-Insensitive Hybrid-Flow Battery. *Acs Energy Lett.* **2**, 411-416 (2017).

5 Gong, K. et al. A zinc–iron redox-flow battery under $100 per kW h of system capital cost. *Energy Environ. Sci.* **8**, 2941-2945 (2015).

6 S. Selverston, z. R. F. S. a. J. S. W. Zinc-Iron Flow Batteries with Common Electrolyte. *J. Electrochem. Soc.* **164**, A1069-A1075 (2017).

7 Xie, C., Duan, Y., Xu, W., Zhang, H. & Li, X. A Low-Cost Neutral Zinc-Iron Flow Battery with High Energy Density for Stationary Energy Storage. *Angew. Chem. Int. Ed.* **56**,14953 –14957 (2017).

8 MCBREEN, J. Rechargeable Zinc Batteries. *J. Electroanal. Chem. Interfa. Electrochem.*168 (1984).

9 Hu, J., Zhang, H., Xu, W., Yuan, Z. & Li, X. Mechanism and transfer behavior of ions in Nafion membranes under alkaline media. *J. Membr. Sci*. **566**, 8-14 (2018).

10 Yuan, Z. et al. Negatively charged nanoporous membrane for a dendrite-free alkaline zinc-based flow battery with long cycle life. *Nat. Commun.* **9**, 3731 (2018).

11 Yuan, Z., Duan, Y., Liu, T., Zhang, H. & Li, X. Toward a Low-Cost Alkaline Zinc-Iron Flow Battery with a Polybenzimidazole Custom Membrane for Stationary Energy Storage. *iScience* **3**, 40-49 (2018).
