# Supplementary material for: Layered double hydroxide membrane with high hydroxide conductivity and ion selectivity for energy storage device
Source: Nat Commun. 2021 Jun 7;12:3409. doi: 10.1038/s41467-021-23721-9 (PMC8184958; doi:10.1038/s41467-021-23721-9)
Supplement: Supplementary file 3 — Description of Additional Supplementary Files [file 41467_2021_23721_MOESM3_ESM.pdf]

Description of Additional Supplementary Files

Supplementary Movie 1: The hydroxide ions transport behavior in LDHs by AIMD simulation.
